# Does entry to center-based childcare affect gut microbial colonization in young infants?

**DOI:** 10.1038/s41598-020-66404-z

**Published:** 2020-06-24

**Authors:** Gerben D. A. Hermes, Henrik A. Eckermann, Willem M. de Vos, Carolina de Weerth

**Affiliations:** 1 Donders Institute for Brain, Cognition and Behavior, Department of Cognitive Neuroscience, Radboud University Medical Center, Nijmegen, The Netherlands; 2 Laboratory of Microbiology, Wageningen University, Wageningen, The Netherlands; 3 Human Microbiome Research Program, Faculty of Medicine, University of Helsinki, Helsinki, Finland

**Keywords:** Microbial communities, Stress and resilience

## Abstract

Entry to center-based childcare (CC) at three months of life can be an important challenge for infants as it includes major stressors such as long maternal separations and frequently changing caregivers. Stress and the new environment may in turn alter the composition of the gut microbiota with possible implications for future health outcomes. As part of an ongoing longitudinal study, we investigated whether CC, as compared to being cared for by the parents at home, alters the composition of the gut microbiota, while accounting for known covariates of the infant gut microbiota. Stool samples of infants who entered CC (n = 49) and control infants (n = 49) were obtained before and four weeks after CC entrance. Using Redundancy analysis, Random Forests and Bayesian linear models we found that infant gut microbiota was not affected in a uniform way by entry to CC. In line with the literature, breastfeeding, birth mode, age, and the presence of siblings were shown to significantly impact the microbial composition.

## Introduction

The human gut microbiota refers to a complex and dynamic population of microorganisms that resides in the human gastrointestinal tract and has recently become object of much scientific endeavors. Intestinal bacteria as part of this ecosystem play central roles in human health and disease^1,2^. They are essential for nutrition, intestinal function, the education of the developing immune system and the protection of the host from invading microbial pathogens^3–5^. Via a bi-directional communication system, intestinal bacteria may even influence brain development and behavior^2,6–8^.

Multiple factors contribute to the development of the human gut microbiota in infancy and the microbial composition becomes relatively stable within the first 3–5 years of life, although some reports describe a longer development phase^9,10^. Because intestinal bacteria influence the development of important host physiological systems within this early and critical developmental time period^11^ it is crucial to understand the factors that influence the establishment of the early gut microbiota. In the present study we will concentrate on an early life factor that can potentially disrupt healthy gut microbial colonization and negatively affect microbial composition: entry into childcare at the age of 3 months. In the following paragraphs, we will outline how gut microbial colonization occurs and why entering childcare at that young age may disrupt this process.

Gut microbiota development is a highly dynamic and individual process. The current consensus is that the first major exposure to microbes happens during the birthing process and is highly dependent on mode of delivery^12–14^. The first inoculation during natural childbirth clearly resembles the maternal fecal microbiota, with potential input from the vagina and other parts of the urogenital tract^14^. In contrast, infants delivered through a Caesarean section (C-section) are colonized with common skin and environmental microbes^12^. Nevertheless, this difference in microbiota composition between children born vaginally or by C-section seems to gradually decrease, although some later life impact has been reported^15,16^.

The initial inoculum initiates a succession of events leading to the development of a child’s own microbiome. In this dynamic process the microbial abundance increases over time, with large fluctuations in the microorganisms present and their relative abundance^17^. Diversity generally increases, aerobes are succeeded by facultative and then strict anaerobes and, roughly up until the introduction of the first solid foods, a well-constrained range of stereotypical bacteria emerge in the faeces. Exclusive breast-feeding generally selects for genera specialized in the utilization of complex human milk oligosaccharides, such as *Bifidobacterium*^18^ and to a lesser extent *Bacteroides* spp, as they can compete for the same ecological niche^19^. By studying both Western and non-Western populations it has been shown that differences exist with regards to community membership, but that the overall temporal dynamics are similar over populations, with aberrant development after C-section delivery, use of antibiotics or early termination of breast-feeding^14,20–23^.

Microbial colonization is characterized by large inter- and intra-individual variability, with large, abrupt, community shifts with interludes of relative stability of varying lengths of time^17,24–26^. Sometimes these shifts occur together with life events that likely instigate considerable environmental pressure, such as antibiotics use, fever, and introduction of formula feeding^26^. Animal models that use maternal separation (MS) to induce early life stress, suggest that early life stress is another such environmental pressure that can induce shifts in the microbiota. MS provokes an adult depressive and anxiety-like phenotype, along with altered immune function, activation of the hypothalamic-pituitary-adrenal (HPA) axis and disruption of the offspring’s microbiota^27–31^. De Palma *et al*. demonstrated the importance of the microbiota to induce these behavioral changes in animals. In germ free mice HPA axis regulation was altered by early life stress, but for the actual induction of the behavioral changes the microbiota was required. This indicated that MS-induced changes in host physiology led to intestinal dysbiosis, which in turn was needed for behavioral changes to occur^32^.

The entrance into center-based childcare (CC) typically starts at three months of life in the Netherlands and includes early life stressors such as long maternal separations, new and frequently changing caregivers and peers, and exposure as well as adaption to a new physical environment. Entering CC produces significant increases in cortisol levels as compared to being cared for in the home environment. Cortisol levels continue to increase until at least a month after entering^33–36^. CC has also been related to other symptoms and illnesses including diarrhea, respiratory illnesses, otitis media, and skin complaints^37^. These findings indicate that entering CC at this young age can be an early life stressor for infants.

This study explores the effects of the entrance to CC on the developing microbiota of 3-month-old infants by comparing infants attending CC to infants being cared for at home. In line with the above-mentioned animal studies, we expected CC to be associated with changes in microbial composition. Specifically, we tested for differences in the relative abundances at a genus-like level (see Methods) as well as differences in alpha diversity by combining univariate Bayesian approaches with multivariate methods including the Random Forests machine learning algorithm that has previously been shown to be useful in microbiome data analyses^21^. We included breastfeeding as a potential protective factor for microbial development, and accounted for known confounders, namely birth mode, antibiotics, age, and the presence of siblings^38^.

## Methods

### Participants

As part of a larger and ongoing longitudinal study (BIBO), 220 mothers were followed since the third trimester of pregnancy, to investigate the influences of early environmental and caregiving factors on child development. Uncomplicated singleton pregnancy, proficiency in the Dutch language, no drug use, and the absence of physical and mental health problems were criteria for initial inclusion. Eight of the 220 women were excluded due to preterm birth or for other medical reasons. In addition, 19 mothers discontinued participation in the study during the first three postpartum months because of personal circumstances. All remaining infants (N = 193) were healthy and born at full term (37 weeks). Infants who had used antibiotics (n = 4) were excluded from the current study. In the first four months of life, mothers collected 9 fecal samples from their infants. Two samples were available for use in this study: Ten weeks post-partum, before entrance to CC (PRE), and 4 weeks after the PRE sample (POST). After eliminating infants who did not provide stool samples for both time points, the final sample size consisted of 49 infants who entered CC (group CC) and 49 infants who were cared for at home (group HOME). Table 1 shows demographic variables for both groups. The age of the HOME group infants was slightly lower than that of the CC group, both for sample PRE (*p* < 0.001) as for sample POST (*p* < 0.001), using Welch’s t-test. There were no significant differences between groups for any other of the shown variables. Within the CC group, infants varied in the number of half-days of childcare per week (Mdn = 4, IQR = 3–4). We tested in a separate analysis whether the number of half-days was associated with gut microbiota composition beyond just the grouping variable, but this effect did not modify the conclusions (Supplementary Table 1). This study and all its experimental protocols were approved by and carried out in accordance with the Ethical Committee of the Faculty of Social Sciences, Radboud University Nijmegen (ECG/AvdK/07.563). Informed consent was obtained from each mother.

**Table 1:** Descriptive statistics for demographic variables of infants and mothers included in the present study.

	CC (n = 49)	HOME (n = 49)	p-value
**Gender**			
Male	29	25	0.417
Female	20	24	
**Age (days) PRE**			
mean (sd)	87.8 $\pm$ 16.0	76.7 $\pm$ 6.3	<0.001
Min	58	68	
Max	123	90	
**Age (days) POST**			
mean (sd)	118.4 $\pm$ 16.1	106.5 $\pm$ 5.9	<0.001
Min	90	97	
Max	154	116	
**Maternal Age (years)**			
mean (sd)	32.9 $\pm$ 3.0	32.2 $\pm$ 3.6	0.306
Min	25.1	24.9	
Max	42.0	40.1	
**Birthweight (grams)**			
mean (sd)	3630.4 $\pm$ 508.9	3636.0 $\pm$ 438.4	0.955
Min	2708	2810	
Max	4600	4700	
**Breastfeeding (Birth - PRE)**			
mean (sd)	5.4 $\pm$ 2.9	5.7 $\pm$ 2.3	0.558
Min	0	0	
Max	11.4	8.9	
**Breastfeeding (PRE - POST)**			
mean (sd)	4.0 $\pm$ 2.9	3.8 $\pm$ 2.8	0.832
Min	0	0	
Max	8.5	8.2	
**Formula-feeding (Birth - PRE)**			
mean (sd)	1.5 $\pm$ 2.4	1.4 $\pm$ 2.1	0.558
Min	0	0	
Max	7.7	7.0	
**Formula-feeding (PRE - POST)**			
mean (sd)	1.8 $\pm$ 2.4	2.0 $\pm$ 2.3	0.832
Min	0	0	
Max	5.9	6.0	
**Proportion breastfeeding (Birth - PRE)**			
mean (sd)	0.8 $\pm$ 0.4	0.8 $\pm$ 0.3	0.558
Min	0	0	
Max	1	1	
**Proportion breastfeeding (PRE - POST)**			
mean (sd)	0.7 $\pm$ 0.5	0.6 $\pm$ 0.5	0.832
Min	0	0	
Max	1	1	
**Siblings**			
Yes	25	32	0.186
No	23	17	
**C-section**			
Yes	6	3	0.294
No	42	45	

*Notes*. CC = center-based childcare. Breastfeeding (Birth - PRE) = average number of breastfeedings per day for the time period between birth and when the first stool sample was obtained. Breastfeeding (PRE - POST) = average number of breastfeedings per day for the time period between the collection of the first and the seconds stool sample. Proportion breastfeeding (Birth - PRE) = proportion of total feedings per day that were breastfeeding for the time period between birth and when the first stool sample was obtained. Breastfeeding (PRE - POST) = proportion of total feedings per day that were breastfeeding for the time period between the collection of the first and the seconds stool sample was obtained. Siblings = presence of siblings at time of birth.

### Microbiota covariates

The mothers received diaries towards the end of their pregnancy with instructions to take weekly notes about breastfeeding and formula-feeding from week 1 until week 27 after birth. For each week, the average number of breast- and/or formula-feedings per day were noted. To determine the effect of breastfeeding we defined two breastfeeding variables: the average number of feedings per day before and during the investigative period (i.e. birth to when the PRE-sample was obtained and PRE to when the POST-sample was obtained). The average number of breastfeedings included the feeding of expressed breastmilk through a bottle. We included the breastfeeding variables, age, siblings and C-section as covariates in all linear models. For 5 infants (4 in CC (8%), 1 in HOME (2%)) breastfeeding data was missing completely. For the univariate analyses the missing values were imputed using multiple imputation (see methods). For the multivariate analysis and visualization the original data was used.

### Feces collection, DNA isolation and microbiota profiling

The parents were instructed to collect the fecal samples at home and to store them at −20 °C. For transportation, samples were kept in coolers and then stored at −20 °C and later at −80 °C before being processed at the Laboratory of Microbiology at Wageningen University. DNA isolation from fecal samples has been described elsewhere in detail^39^. In brief, DNA was isolated using a combination of column purification and Repeated-Bead-Beating. Purity and concentration of DNA were assessed with a Nanodrop 1000 spectrophotometer (Thermo Fisher Scientific, Wilmington, USA). The analysis was then performed utilizing a previously benchmarked custom made, phylogenetic microarray, the Human Intestinal Tract Chip (HITChip)^40,41^. The HITChip contains a duplicated set of 3,631 probes, which target the V1 and V6 hypervariable regions of the 16 S rRNA gene of 1140 intestinal bacterial phylotypes. After extraction of DNA, the full-length 16S rRNA gene was amplified by PCR using primers T7prom-Bact-27-for and Uni-1492-rev^41^. This was followed by *in vitro* transcription and labelling of the resulting RNA with Cy3/Cy5 before hybridization to the array. The signal intensity data from the microarray hybridizations were collected from the Agilent G2505C scanner (Agilent Technologies) using the Agilent Feature Extraction software, version 10.7.3.1 and pre-processed using an in-house MySQL database and custom R scripts. Each scanner channel from the array was spatially normalized separately using polynomial regression, followed by outlier detection and filtering in each set of probes with a χ2 test. Each sample was hybridized at least twice to ensure reproducibility. Duplicate hybridizations with a Pearson correlation <0.95 were not considered for further analysis. Microbiota profiles were summarized to genus-like 16S rRNA gene sequence groups with a sequence similarity >90% referred to as species and relatives (‘et rel.’). Measurements of probes that belong to the same phylotype were normalized with Robust Probabilistic Averaging^42,43^. Log10-transformed hybridization signals were used as a proxy for bacterial abundance.

### Statistical analyses

#### Microbiota analysis

All analyses were performed in R version 3.5^44^. Bacterial richness was calculated at the probe level by using an 80% quantile threshold for detection of each individual probe. Diversity within a sample is termed alpha diversity and was calculated using the Shannon metric. HITChip signals were transformed to relative abundance. To determine the dynamics of microbial groups we calculated their coefficient of variation (CoV). CoV is a standardized measure of dispersion defined as the ratio of the standard deviation ($\sigma$) to the mean (µ). For the redundancy analysis (RDA) and the Bayesian robust linear models we applied centered-log-ratio (clr) transformation^45^. The clr-transformation of relative abundances allows for the application of statistical methods that have been developed for real random variables, such as RDA and the Bayesian models^46^. To determine the multivariate effects of CC and the number of half-days in CC on overall microbiota composition, we performed redundancy analysis (RDA) while accounting for the following variables that are known to influence microbiota composition: breastfeeding (average number of feedings during and before the investigative period), birth mode (C-section vs natural birth), age in days, and the presence of siblings using the function *rda* from the vegan package^47^.

To determine the univariate effects of CC entry on alpha diversity and each bacterial group individually, we performed Bayesian hierarchical robust linear models as described by Krushke *et al*.^48^. Bayesian data analysis provides several advantages over classical null hypothesis group comparison methods. These include richer information about parameter estimates, as the method provides complete distributional information about model parameters such as means and standard deviations, including credibility intervals of all possible combinations of these parameters. Furthermore, Bayesian data analysis delivers more precise information about the uncertainty when estimating group differences. The robust linear model presented by Krushke *et al*. in particular has advantages over standard linear models: The model is able to accurately estimate the mean (${\boldsymbol{\mu }})$ and standard deviation (${\boldsymbol{\sigma }})$ when outliers are present as it utilizes the student t-distribution instead of the gaussian distribution. Furthermore, standard linear models assume homogeneity of variance between groups while this assumption is often violated in the context of differential abundance testing^48^. In addition, an environmental factor could lead to a change in the variance of a distribution rather than (only) a change in the mean. This possibility is not considered in standard models. Our model allows the standard deviation ${\boldsymbol{\sigma }}$ to vary between the groups by modeling it as a linear function of the grouping variables CC, time, siblings and birth mode. The model can be written as:

$${y}_{i}\sim T({\boldsymbol{\nu }},{\boldsymbol{\mu }},{\boldsymbol{\sigma }})$$

where

$$\begin{array}{lll}{\mu }_{i} & = & {\beta }_{0i[j]}+{\beta }_{1}\times CC+{\beta }_{2}\times time+{\beta }_{3}\times CC\times time+{\beta }_{4}\times age+{\beta }_{5}\times bf+{\beta }_{6}\\  &  & \times \,sib+{\beta }_{7}\times csec+{\beta }_{8}\times sib\times csec\,{\rm{and}}\,{\sigma }_{i}={\beta }_{\sigma 0}+{\beta }_{\sigma 1}\times CC+{\beta }_{\sigma 2}\times time\\  &  & +\,{\beta }_{\sigma 3}\times CC\times time+{\beta }_{\sigma 4}\times sib+{\beta }_{\sigma 5}\times csec++{\beta }_{\sigma 6}\times sib\times csec\end{array}$$

Besides ${\boldsymbol{\mu }}$ (mean) and ${\boldsymbol{\sigma }}$ (standard deviation (SD)), the model consists of the parameter ${\boldsymbol{\nu }}$, which represents the normality parameter (low values lead to long and heavy tails, whereas as ${\boldsymbol{\nu }}$ increases, the distribution approaches the gaussian distribution). The *j* in ${\beta }_{0i[j]}$ indicates that each subject can deviate from the overall mean (often referred to as partial pooling or mixed effects modeling).

The goal is to infer differences in $\mu$ and $\sigma$ of the assumed distribution of relative bacterial abundances between the subgroups (CC PRE, CC POST, HOME PRE and HOME POST), while accounting for the effects of the other environmental variables. This implies multiple comparisons of model parameters. In the Bayesian framework we can use a normal prior centered at 0 with a standard deviation of 1 for each comparison of interest across all the models so that the Bayesian 95% credible interval (CI) of the effect size is always more likely to include zero compared to the classical confidence interval, which makes Bayesian inference a more conservative approach^49^. The robust linear models were fitted using the package *brms*, which uses the probabilistic programming language Stan^50,51^. Stan utilizes Hamilton Monte Carlo (HMC), a Markov chain Monte Carlo (MCMC) method, to estimate parameters. To ensure proper convergence of the chains, we investigated individual chains using Shinystan and screened diagnostic parameters (divergent transitions and rhat values)^52^. To visualize the posterior predictive intervals we utilized the tidybayes package^53^. Brms allows multiple data sets as input, which enables the use of multiple imputation. We used predictive mean matching (PMM) as implemented in the *mice* package^54^ to impute the missing data points within the breastfeeding variable. PMM is robust against misspecification of the imputation model while performing as well or better than common parametric imputation models under various missingness conditions with up to 20–30% missing values^55,56^. In contrast, the variable breastfeeding had 10% missing. The multiple imputation resulted in 10 new datasets that were used to fit the Bayesian models. We also explored the same models using listwise deletion.

Finally, to evaluate whether we could predict childcare entrance based on microbiota composition after one month, we used the Random Forests machine learning algorithm (RF) with relative abundance data. RF is a tree-based ensemble learning method that is well suited for classification based on microbial abundances^57^. We performed repeated 10-fold cross-validation (10 × 10) to estimate the classification accuracy using the caret package^58^.

## Results

### Infant microbiota composition and dynamics

**Figure 1:**
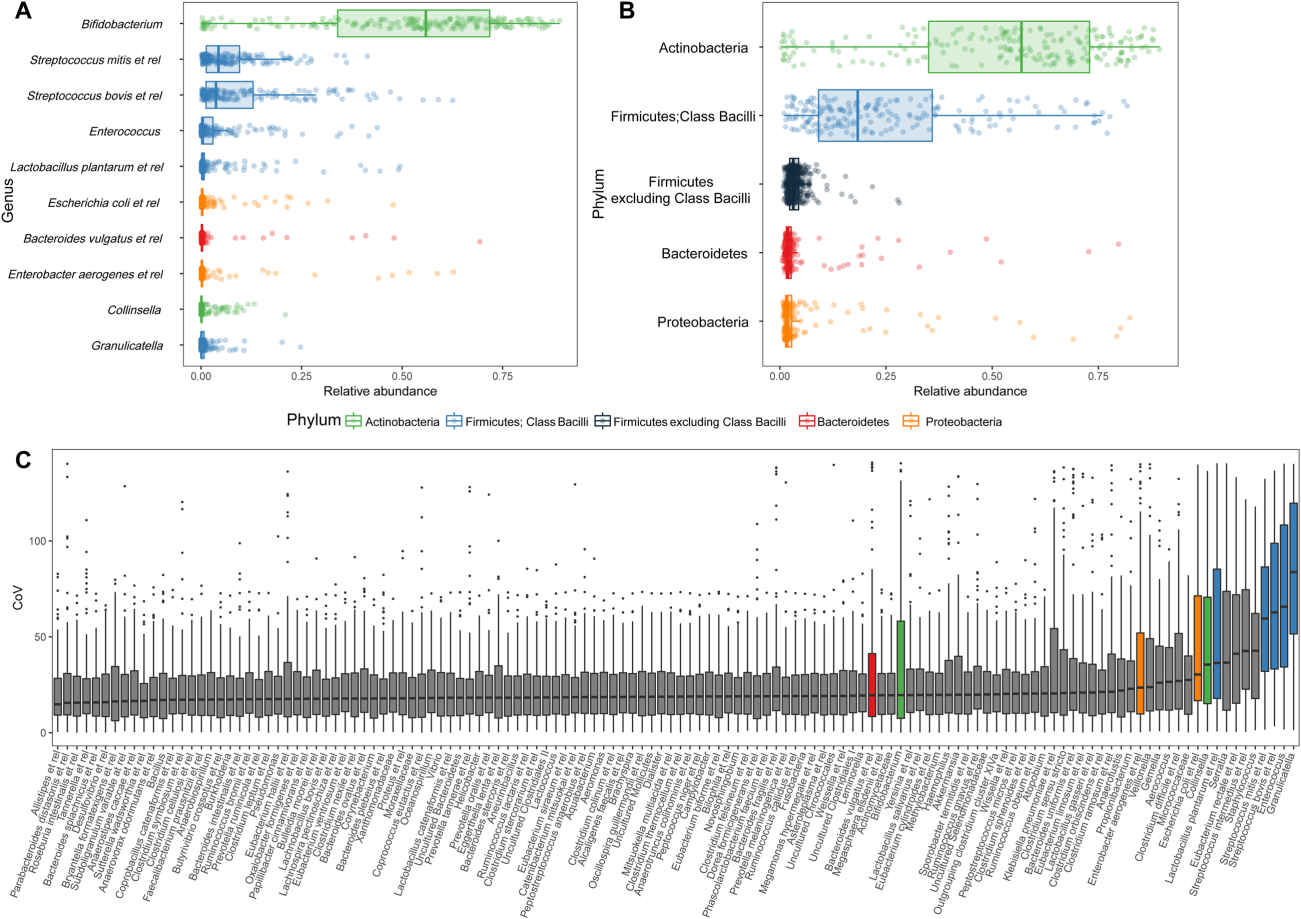
Infant microbiota composition and dynamics. (**A**,**B**) Relative abundance of major genus-like groups and their Phyla with mean abundance of >1% at the population level. (**C**) Coefficient of Variation (CoV) of all genus-like groups. The infant microbiota is highly variable as it is dominated by only a few taxa, which are also among the most variable. GREEN – Actinobacteria; BLUE – Firmicutes, Class Bacilli; BLACK – Firmicutes excluding Class Bacilli; RED – Bacteroidetes; ORANGE – Proteobacteria.

Overall, the infant microbiota was dominated by only a few genera from four Phyla; Actinobacteria, Firmicutes (specifically members from the Class Bacilli), Bacteroidetes and Proteobacteria. At the genus level, these were *Bifidobacterium* spp. with a mean relative abundance of 51%, followed by facultative anaerobes from the Bacilli (bacteria related to *Streptococcus* (16.8%), *Enterococcus (3.4%)*, *Lactobacillus plantarum* et rel (2.9%) and *Granulicatella (1.0%*)). The cumulative mean relative abundance of these groups was more than 75%. Additionally, the variation in relative abundance of these taxa at the population level was very high. For instance, the relative abundance of *Bifidobacterium* spp. ranged from completely dominating (89%) to almost absent (0.2%) (Fig. 1A,B). Apart from being the most dominant taxa at the population level these were also among the most variable within subjects as determined by their CoV (Fig. 1C). The fact that the most abundant taxa display the highest variability implies a highly variable microbiota.

### Effects of childcare entry on infant microbiota

#### Redundancy analysis of the effects of childcare entry on infant gut microbiota composition

We determined whether childcare (CC) entry affected overall microbiota community composition using Redundancy Analysis (RDA). RDA is a direct gradient analysis technique which summarizes linear relationships between components of response variables (microbiota) explained by a set of explanatory variables (CC and covariates) by multiple linear regression of the multiple response variables on the multiple explanatory variables. To determine how the different environmental variables interact with and impact the microbiota, we calculated their simple effects (i.e. the effect of the environmental variable on the microbiota without any other covariates) as well as the conditional effects (the impact on the microbiota when the effect of the other variables are partialled out). This allowed us to determine the effect of each variable on its own, but also their combined effects.

**Figure 2:**
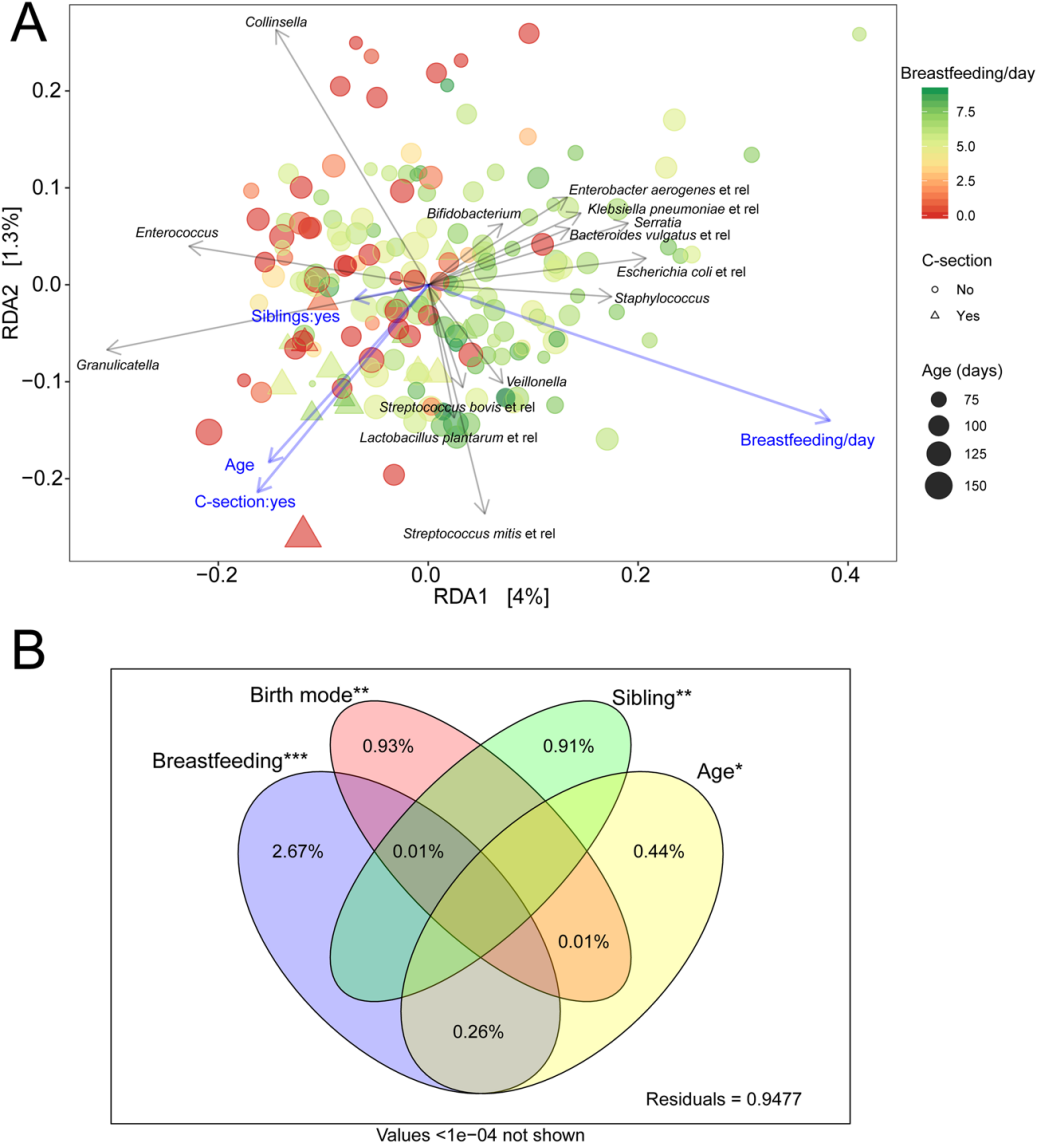
The impact of birth mode, breastfeeding, siblings and age on the infant gut microbiota. (**A**) Redundancy analysis (RDA) visualizing microbiota composition of all fecal samples (n = 196) colored by the number of breast-feedings and the size of the points scaled by age in days. Individuals born by C-section are represented as triangles. RDA displays and explains the variation explained in the microbiota, constrained by the predictor variables. Blue arrows depict the significant environmental variables and grey arrows the abundance of bacterial groups. Length of the arrows is a measure of fit. The longer the arrow the higher the association. (**B**) Venn diagram visualizing the partitioning of the variation explained by the significant predictors. ***P < 0.001, **P < 0.01, *P < 0.05.

We did not find a significant effect of CC entry or the number of half-days in CC (Supplementary Table 1), compared to staying at home, on the microbiota. Neither in separation nor combined with other environmental variables. Nevertheless, birth mode, feeding mode, age, and siblings, were significantly correlated to the microbiota in concordance with literature^38^. The strongest effect was from breastfeeding, with decreasing effect sizes for birth mode, siblings and age. All simple and conditional effects and corresponding p-values and their respective effect sizes (R^2^) are shown in Table 2. R^2^ reflects the percentage of variation explained out of the total microbiota variation; i.e. a higher R^2^ implies a stronger effect. All these findings are combined in a tri-plot visualizing the relation of the environmental variables with each other and their resulting effect on the microbiota (Fig. 2A). The relation between the variation explained by the environmental variables and their overlapping conditional effects is visualized in a Venn diagram (Fig. 2B).

**Table 2:** RDA models output.

	Df	Variance	F	Pr (>F)	R^2^
**Simple effects**					
Time	1	0.317	1.4265	0.129	0.008
CC	1	0.274	1.2314	0.256	0.007
Age	1	0.436	1.9677	0.028*	0.011
Sibling	1	0.456	2.0602	0.019*	0.011
Birth-mode	1	0.559	2.5288	0.015**	0.014
Breastfeeding	2	1.605	3.7085	0.001***	0.032
CC × Time	3	0.781	1.172	0.226	0.019
**Conditional effects**					
Age	1	0.436	2.0652	0.018*	0.004
Sibling	1	0.598	2.8338	0.003**	0.010
Birth-mode	1	0.609	2.8843	0.002**	0.010
Breastfeeding	2	1.663	3.9364	0.001***	0.027

#### Bayesian group comparisons of effects of entry on individual microbial groups and microbiota diversity

To gain more insight on the association of CC entry and other environmental variables with individual bacterial groups and microbiota diversity, we performed Bayesian hierarchical robust linear models. Bayesian approaches provide more detailed information about the uncertainty when estimating parameters such as group differences or slope parameters in linear models. The robust linear model as described by Kruschke *et al*. is particularly well suited to model distributions when outliers are present and to address common model violations in standard linear models such as heterogeneity of variance^48^. In the following, for covariates, *effect* refers to the magnitude of the slopes whereas for the group comparisons *effect* refers to the difference in the means. To compute the difference in means between two groups (e.g. CC-PRE – CC-POST) the calculated posterior distributions of their means are subtracted. We make a statement with confidence about the effect size being larger than zero when 95% of the posterior distribution excludes zero. The use of listwise deletion instead of multiple imputation led to similar results.

**Figure 3:**
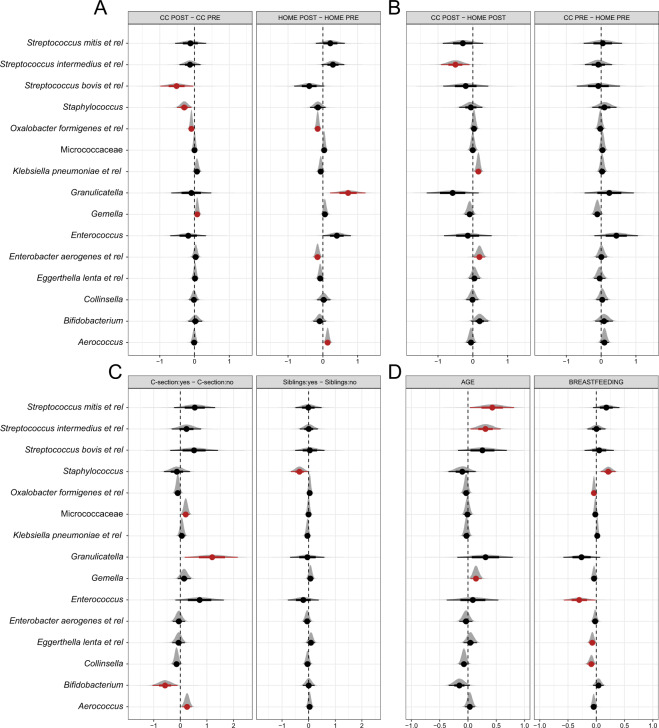
Bayesian hierarchical robust linear model posterior distributions for individual bacterial group differences. (**A**) within group effects, (**B**) between group effects and (**C**) C-section and siblings (**D**) age and breastfeeding. For the covariates (**D**) the x-axis refers to the magnitude of the slopes whereas for the group comparisons (**A**–**C**) it refers to the difference in the means. Taxa are shown in red when the probability that the absolute effect size is >0 exceeds 0.95, given our model and the data.

Figure 3 shows the posterior distributions of interest whereby red coloring indicates that we can make a claim with confidence. Within the CC or HOME group (Fig. 3A), temporal effects were as follows. Within CC, bacteria related to *Streptococcus bovis* and *Staphylococcus* were lower after one month, whereas within HOME bacteria related to *Granulicatella* and *Aerococcus* were higher, while those related to *Enterobacter aerogenes* and *Oxalobacter formigenes* were lower. We did not detect differences in relative bacterial abundances between the two groups (Fig. 3B, right graph) before CC entry, while after one month the relative abundance of the Proteobacteria related to *Enterobacter aerogenes* and *Klebsiella pneumoniae* was lower in the HOME group, while that of *Streptococcus intermedius* was higher (Fig. 3B, left graph).

Figure 3C shows that infants born via C-section showed higher relative abundances of bacteria related to *Granulicatella*, *Aerococcus* and Micrococcaceae, but lower relative abundances of *Bifidobacterium*. Infants with siblings were found to have lower levels of *Staphylococcus* and also a lower SD of this taxon. A lower SD was also confirmed for *Enterococcus*, without differences in the mean. Finally, as can be seen in Fig. 3D, a higher number of daily breast-feedings was associated with more bacteria related to *Staphylococcus* and less to *Enterococcus*, *Collinsella*, *Eggerthella lenta* and *Oxalobacter formigenes*. A higher age was positively associated with bacteria related to *Streptococcus mitis*, *Streptococcus intermedius* and *Gemella*.

**Figure 4:**
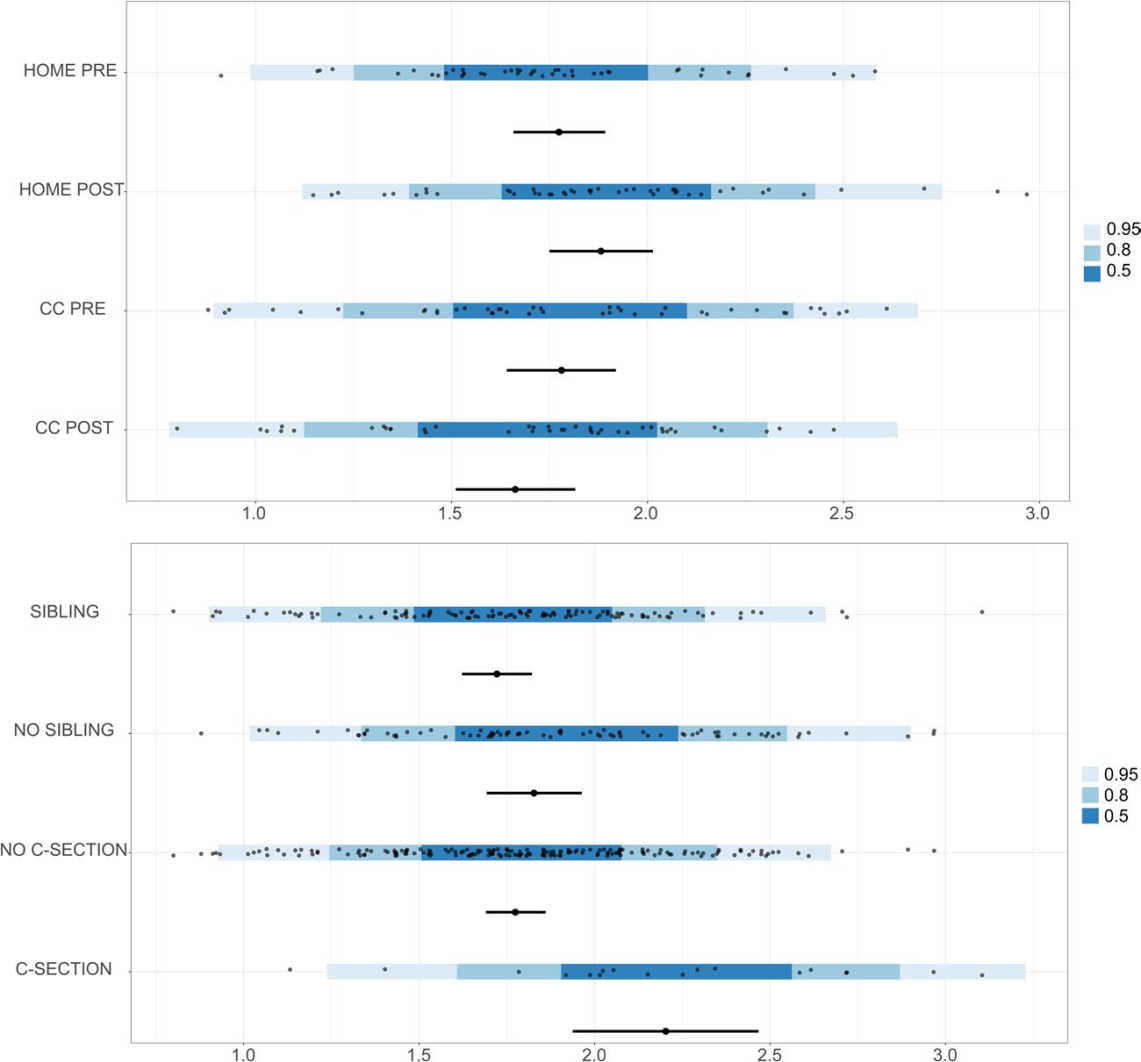
Bayesian hierarchical robust linear model group posteriors for microbiota alpha diversity. Means of Shannon diversity per group with 95% CI are shown with the black point range. The observed values are shown as black points within the blue bar. The Bayesian posterior predictive intervals are shown in blue. The predictions were made using the median for average number of breast-feedings and median age at PRE and POST.

We used the same approach to compare microbiota diversity between groups. Table 3 shows the estimated difference in the means and standard deviations between the groups as well as the magnitude of the slopes for the covariates. Within the CC or HOME groups our model estimates that there is no temporal effect on diversity as well as between the two groups before CC entrance. However, when comparing HOME and CC one month after entrance, the average diversity was estimated to be lower in the CC group. Figure 4 shows the means of the Shannon diversity index per group with 95% CI (black point range) as well as the observed values (black points) and the posterior predictive interval (blue bar). To calculate the predictive interval, we used the median for the average number of breast-feedings and the median age at PRE and POST, respectively. The Bayesian predictive intervals illustrate the uncertainty of the predictions: the estimated distributions for the CC/HOME groups are very much overlapping despite the mean difference, whereas C-Section seems to have a stronger impact on diversity. Age and breastfeeding did not reach our predefined threshold to make a statement with confidence about the effect being >0 and there was no difference in the estimated standard deviations between groups.

**Table 3:** Estimated model parameters for microbiota diversity (Shannon).

Comparison	Median	95% CI
***Difference in means***		
CC PRE - HOME PRE	0.01	[−0.18, 0.19]
HOME POST – HOME PRE	0.11	[−0.01, 0.23]
CC POST – CC PRE	−0.12	[−0.26, 0.03]
CC POST – HOME POST	−0.22	[−0.41, −0.03]
***Covariates***		
C-section:yes – C-section:no	0.43	[0.15, 0.7]
Sibling:yes – Sibling:no	−0.11	[−0.27, 0.06]
Age	0.06	[−0.07, 0.2]
Breastfeeding	−0.06	[−0.13, 0.01]
***Difference in SD***		
CC PRE – HOME PRE	0.07	[−0.02, 0.17]
HOME POST – HOME PRE	0.01	[−0.09, 0.1]
CC POST – CC PRE	0.02	[−0.13, 0.19]
CC POST – HOME POST	0.08	[−0.04, 0.22]
C-section:yes – C-section:no	0.06	[−0.06, 0.24]
Sibling:yes – Sibling:no	−0.02	[−0.12, 0.07]

#### Random Forest analysis of non-linear relationships between childcare entry and microbiota composition

Finally, we used the random forests (RF) algorithm to determine if we could accurately classify whether an infant belonged to the HOME or CC group after one month. The latter would indicate a characteristic effect of CC on the infant gut microbiota. The benefit RF has over the linear models is its ability to detect non-linear associations. The prediction accuracy using repeated cross validation was close to random classification with 53.5%, suggesting that CC did not produce a strong uniform shift in microbiota composition.

## Discussion

This study examined the effect of entry into center-based childcare (CC) on gut microbial composition of 3–4-month-old infants by comparing the microbial composition between infants that entered CC at 3 months and infants that were cared for at home (HOME) at that age. For all infants, we assessed microbial composition at two time points: At 10 weeks of age (PRE), which was before CC entrance for the CC group, and 4 weeks later, or 4 weeks after entrance for the CC group (POST). We accounted for known covariates including age, presence of siblings (yes vs no), delivery mode (natural vs C-section) and the average number of breast-feedings per day in the period before measurement of the microbiota. We combined multivariate (Redundancy analysis and Random Forest algorithm) with Bayesian univariate statistical methods to test our hypothesis that CC entrance is associated with changes in microbial composition over time.

The microbiota of all the infants exhibited a low microbial diversity as it was dominated by only a few typical bacterial groups from the phylum Actinobacteria (*Bifidobacterium* spp and *Collinsella*), facultative anaerobes from the Firmicutes (such as *Streptococcus* spp, *Lactobacillus* spp and *Enterococcus* spp), Proteobacteria (*E.coli* and bacteria related to *Enterobacter aerogenes*) and Bacteroidetes (*Bacteroides* spp*)*. These findings are in line with findings from previous studies in the US and Europe (Germany, Finland, Sweden and the Netherlands)^38,59,60^.

In contrast to breastfeeding, birth mode, age, and the presence of siblings, CC was not associated with gut microbiota composition according to Redundancy analysis (RDA). In line with that, we could not achieve higher accuracy than by chance using the Random Forest algorithm to classify CC vs HOME using the POST samples. Bayesian univariate analyses, that also take the individuality of the starting microbiota into account, did show a few taxa to be differently distributed between the two groups in the POST samples. In the HOME group the relative abundances of Proteobacteria related to *Enterobacter aerogenes* and *Klebsiella pneumoniae* were lower while that of *Streptococcus intermedius* was higher compared to the CC group at time POST. Except for bacteria related to *Streptococcus intermedius*, these bacterial groups were also heavily influenced by other environmental variables. Using the same Bayesian approach, we observed a lower Shannon alpha diversity in the CC group compared to the HOME group at time POST. All in all, our results show that entrance to CC does not result in a complete and homogeneous ‘disruption’ or ‘dysbiosis’ of the microbiota as reported for rodents subjected to early life stress.

There are several possible explanations for the lack of a general effect of the entrance to childcare on infants’ gut microbiota. First, although CC entrance may be considered a major stressor in a young infant’s life that is accompanied by substantial rises in stress hormones^33–36^, it might not be comparable in severity to maternal separations in rodents. E.g. in our CC sample infants were taken care of by surrogate caregivers, while in maternal separation paradigms with rodent pups, there is no surrogate caregiver during the separation^27^. Also, Dutch infants typically attend childcare for around 2 days a week^61^; this contrasts with practices elsewhere in the world and may not be enough exposure to produce major effects on the microbiota. Second, rodents lack the genotypical variation found in human populations and are kept and studied in controlled laboratory environments in which the individual variation in gut microbiota is small^62,63^. Hence, in rodent models the effects of environmental pressures can be studied in complete isolation. This may lead to a much greater response to an individual stressor than it normally would when other (stronger) environmental drivers of microbiota composition are present. Finally, the infant gut microbiota at 3–4 months of age is still in an unstable, dynamic, highly individual developmental stage^17,24–26,38^. This was confirmed by a very large intra and inter-individual microbiota variability in our population. Microbiota at these young ages appears to show large fluctuations, probably resulting from a myriad of environmental influences. Additionally, infants of the present study went to different childcare centers, which exposed them to different built environments, caregivers and other infants with different microbiomes, thereby increasing the variety of environmental influences on the microbiota. Nevertheless, a generalizable stress-related effect across centers would most likely have been detectable, despite these (unknown) and potential center-specific confounding variables. Other factors than those we controlled for are e.g. fever, contact with animals, and/or genetics^26,38^. The instability of the infant microbiota might also possibly be an intrinsic property of the dynamics of the gut microbial colonization and may obscure the effects of individual environmental factors that may each have only a modest influence on gut microbiota composition. In other words, an environmental factor would need to be very strong to generate a universal disruption in the microbial composition that overrides the normally occurring fluctuations in infant gut microbiota in the first months of life.

The results of our study showed significant associations with known important environmental factors. Their effects were often partly overlapping and impacted similar bacterial groups (Fig. 2A,B), possibly indicating an accelerated colonization process in infants born by C-section. For instance, being older and being born by C-section were both positively associated with the abundance of the highly variable facultative anaerobes from the Bacilli and related to *S. bovis* and *S. mitis L*. *plantarum* and *Granulicatella* at the expense of *Bifidobacterium* and several Proteobacteria. Contrarily, a younger age and being born vaginally were both associated with higher relative abundances of *Bifidobacterium*, *Collinsella* and several Proteobacteria in line with previous research of (Dutch) infants^59,64^. The latter pattern, except for *Collinsella*, was also associated with having no siblings. C-section delivery was associated with higher Shannon diversity. This increase in diversity may be the result of the lower proportions of the generally dominant *Bifidobacterium* in this group of infants, as has been previously reported^65^.

Breastfeeding showed the strongest association with infant gut microbiota composition. However, surprisingly, breastfeeding was only weakly related to *Bifidobacterium*, but was rather more associated with increases in mainly *Staphylococcus* and to a lesser extent, Proteobacteria (*Serratia*, *E. coli, Klebsiella pneumoniae* and *Enterobacter aerogenes* et rel) and *Bacteroides vulgatus* et rel. Breastfeeding was also strongly associated with a decrease in *Enterococcus* and *Collinsella*. In recent years in European countries, infant formulae have often been supplemented with prebiotics such as short chain galacto-oligosaccharides (scGOS) alone, or in a mixture with a chicory root derived inulin containing long chain fructo-oligosaccharides (lcFOS)^66^. Prebiotics mimic the bifidogenic effect of oligosaccharides found in human milk thus preventing the difference in *Bifidobacterium* abundance that was previously associated with formula feeding^60^. Furthermore, human milk itself has been found to contain bacteria, including Proteobacteria, as well as *Staphyloccocus*, which is generally associated with the skin^67^. The latter has also been previously found to be depleted in formula-fed infants in concordance with our earlier findings^60^. In total, the environmental variables explained a non-redundant ~5.9% of the microbiota composition. This is in the same order of magnitude as previously reported in other human studies^68^. This means that in infants generally only a relatively small proportion of the gut microbial composition can be explained by the factors that are most commonly accounted for.

Despite the fact that we did not find an effect of CC entrance on the microbiota at the group level even after using different statistical approaches, it is not possible to conclude that there was no disrupting effect on the microbiota at the individual level. Individual infants did show large changes in the microbiota between the two assessments, but these changes were not uniform across individuals. Although temporal microbial dynamics at the population level, with regards to bacterial succession patterns, have been shown to be universal over different cultures and geography^59^ it is known that this process is very variable between individuals^17,24–26^. Future studies including larger populations and especially repeated measurements from before and after life changing events are necessary to determine whether the resulting bacterial shift is typical or deviant for a specific individual. Subsequently, potential sub-groups of infants displaying specific signatures of dysbiosis can be determined, and their microbial signatures related to possible adverse health outcomes later in life. However, given the importance of gut microbial colonization for the development of the microbiota-gut-brain axis and the future health of the individual^11^, it is also possible that the colonization process might be robust against perturbations such as entrance to CC, hence explaining the lack of microbial change at the group level.

This is the first human study that examined the effect of entrance to center-based childcare on microbiome development. The strengths are that it included a relatively large population of healthy infants with a natural variation of environmental variables and an ecologically valid stressor as opposed to typical rodent studies that generally include low number of individuals under strictly controlled laboratory conditions. Another strength is that we used a combination of different multivariate and Bayesian univariate statistical methods to analyze the data. However, there are also some weaknesses with regards to this study. Our population was ethnically and socio-economically uniform^69^. This can be seen as an advantage, but it precludes generalization to the larger population. Also, to conclude whether the microbiota is disrupted at an individual level, more sample time points than just PRE and POST would be needed. For example, it would be interesting to follow infants entering childcare for a longer period of time, as elevated infant cortisol has been observed throughout the first months in childcare^34^. Larger and more stable differences in the gut microbiota of infants attending childcare and those being taken care of at home, may only appear after a longer period of time in childcare.

## Conclusion

Entering center-based childcare has been shown to produce large increases in stress hormones in infants and can therefore be considered a significant stressor in early life. Childcare includes maternal separation and in animal models early life maternal separation has been found to lead to large shifts in gut microbial composition. However, in the present study infant gut microbiota was not impacted in a uniform way by entering childcare at the age of 3 months. Large shifts in gut microbiota were observed, but were idiosyncratic to individual infants and were also observed in infants not attending center-based childcare. In general, the infants’ gut microbiota was found to be intrinsically very dynamic. Other environmental variables, namely breastfeeding, birth mode, age, and the presence of siblings, were shown to significantly impact the microbial composition, with effects that were largely overlapping and typically included the most abundant and variable taxa. Our results suggest that in infants the stress-inducing effects of childcare entry might not be as strong as the maternal separation paradigms of animal models. Alternatively, general effects may potentially only become visible after longer periods of childcare entry and when infants are older and their gut microbiota has become more stable.

## Supplementary information

Supplementary information.
