# Supplementary material for: Does entry to center-based childcare affect gut microbial colonization in young infants?
Source: Sci Rep. 2020 Jun 24;10:10235. doi: 10.1038/s41598-020-66404-z (PMC7314774; doi:10.1038/s41598-020-66404-z)
Supplement: Supplementary file 1 — Supplementary information. [file 41598_2020_66404_MOESM1_ESM.docx]

**Supplementary table 1.**

**Does entry to center-based childcare affect gut microbial colonization in young infants?**

Gerben D. A. Hermes^2,†^, Henrik A. Eckermann^1,†^, Willem M. de Vos^2,3^ , Carolina de Weerth^1,*^

**Supplementary table 1.** RDA models output with Childcare attendance as half days instead of as a grouping factor.

| Model Parameter | Sum of Squares | Mean Sum of Squares | F | Df | p | R^2^ |
| --- | --- | --- | --- | --- | --- | --- |
| Childcare | 43.52 | 43.525 | 1.096 | 1.00 | 0.334 | 0.006 |
| Time | 61.44 | 61.441 | 1.548 | 1.00 | 0.082 | 0.008 |
| Breastfeeding | 204.80 | 204.797 | 5.158 | 1.00 | 0.001 | 0.027 |
| Age | 62.19 | 62.186 | 1.566 | 1.00 | 0.113 | 0.008 |
| Childcare:Time | 29.75 | 29.751 | 0.749 | 1.00 | 0.691 | 0.004 |
| Residuals | 7,305.03 | 39.701 | - | 184.00 | - | 0.948 |
| Total | 7,706.73 | - | - | 189.00 | - | 1.00 |
